# Editorial: Reproductive medical care in minors: ethical and liability issues

**DOI:** 10.3389/fmed.2024.1378211

**Published:** 2024-02-27

**Authors:** Mariano Cingolani, Vittoradolfo Tambone

**Affiliations:** ^1^Department of Law, Institute of Legal Medicine, University of Macerata, Macerata, Italy; ^2^Research Unit in Bioethics and Humanities, Campus Bio-Medico University, Rome, Italy

**Keywords:** human reproduction, informed consent, confidentiality, protection, medical ethics, minors

The proposal for the Research Topic focuses on the study of responsibility and ethical aspects related to health workers involved in assisting minors in their reproductive health. It addresses legal and ethical issues concerning the minors exercise of their right to assistance, their autonomy, their ability to comprehend risks, and make informed choices. The attention was also delves into the responsibility of healthcare professionals in assisting minors in their reproductive health, including invasive procedures and drug therapies.

The WHO already emphasized in 2010 that “Adolescence is not an easy time. Young people can be vulnerable, at risk of low self-esteem, unwanted pregnancies, induced abortions, and sexually transmitted infections. Just one raised eyebrow or unfriendly comment can deter young people from seeking help with sexual and reproductive health concerns, sometimes with serious consequences. They may feel that only internet blogs and chat rooms can provide assistance^1^.” Even though a 2021 survey reports a 17% decrease in sexual activity among adolescents, the importance of the topic remains unchanged and highly relevant.

The goal was to explore the ethical dilemmas arising from such practices and propose appropriate solutions that consider fundamental ethical principles and the rights of minors. This includes considerations related to any interventions in the decisions made by parents. The contributions also addressed aspects connected with the legal regulation of interventions on the reproductive health of minors and the corresponding responsibilities of healthcare professionals in adhering to these regulations, encompassing both pharmacological and operational approaches.

The Research Topic features six articles, comprising 2 original research articles, 1 review, 1 opinion piece, 1 brief research report, and 1 perspective. Each of these contributions addresses specific aspects of reproductive medicine applied to minors, aligning perfectly with the objectives of the Research Topic.

The two original research contributions concern the evaluation of medication errors in nursing during the COVID-19 pandemic and their relationship with shift work at teaching hospital (*Evaluation of medication errors in nursing during the COVID-19 pandemic and their relationship with shift work at teaching hospitals: a cross-sectional study in Iran*, by Gilavand et al.) and the use of telomere-related genes as potential biomarkers predictive of endometriosis (*Telomere-related genes as potential biomarkers to predict endometriosis and immune response: Development of a machine learning-based risk model*, by Zhang et al.).

The article by Gilavand et al. is structured as a cross-sectional study about the evaluation of relationship beetween shift work and medication errors in nursing during the COVID-19 pandemic at teaching hospitals. The study involved 260 nurses and utilized three questionnaires [demographic characteristics questionnaire; medication error questionnaire; standard Circadian Type Inventory (CTI)]. Resulted that 36.2% of nurses reported a medication error during the COVID-19 pandemic, with of which (65.8%) occurring during the night shift. Between medication errors and shift work was noticed a significant relationship. Medicating one patient's drug to another (28.84%) and giving the wrong dose of drugs (27.69%) were the most common types of medication errors. The highest rates of medication errors were in Emergency wards, in pediatric and gynecology situations. The conclusions of the study was that medication errors increased due to shift work and the COVID-19 pandemic. The article recommends, particularly in critical situations, implementing plans to prevent burnout and to reduce fatigue and anxiety among nurses. To establish reporting systems, to implement error reduction strategies, to effort the identify risky areas, can contribute to the development of preventive medicine, even in reproductive medicine for minors.

The article by Zhang et al. is structured as a machine-learning-based risk model to study telomere-related genes as potential biomarkers to predict endometriosis, an aggressive, pleomorphic, and common gynecological disease, also present in adolescents and young women. Telomere-related genes (TRGs) were obtained from TelNet, and RNA-sequencing (RNA-seq) data of endometriosis patients were extracted from three datasets in the GEO database. To identify telomere signature genes and build nomogram prediction models a random forest approach was used. To identify the pathways involved in the action of the signature genes Gene Ontology, Kyoto Encyclopedia of Genes and Genomes, and Gene Set Enrichment Analysis were employed. Fifteen genes were identified as endometriosis–telomere differentially expressed genes, and machine learning further narrowed it down to six characteristic predictive genes for endometriosis. The results indicate that telomere-related genes play an important role in the development of endometriosis and are associated with immune infiltration, acting on multiple pathways, including the cell cycle. Even in adolescents and young women telomere signature genes can represent a predictive markers for endometriosis.

The article published in Research Topic as review concerns an algorithm to improve diagnosis and treatment of vaginitis (*Diagnosis and treatment of infectious vaginitis: Proposal for a new algorithm, by* Eleutério et al.). In January 2022 was conducted a preliminary literature research within biomedical databases PubMed and SCieLo utilizing the following search terms: “bacterial vaginosis” and/or “vaginitis” AND “clinical reasoning,” “differential diagnosis,” “diagnostic,” “treatment,” and “approach.” The selected articles were assessed by three experienced researchers to identify the main data and develop algorithms. Detailed algorithms were created with the primary goal of enhancing gynecological practice by considering various scenarios and access to diagnostic tools, ranging from the simplest to the most complex tests. Different age groups and specific contexts were also taken into account. The combination of anamnesis, gynecological examination, and complementary tests remains the foundation for a proper diagnostic and therapeutic approach. Regular updates of these algorithms are essential as new evidence becomes available. The produced algorithms are valuable for improving the diagnosis and treatment of vaginitis, even in pediatric and juvenile women.

The article published as brief research report reports a casuistic related to six cases of civil litigations consecutive to applications of procedure of female sterilization (*Legal medicine aspects of female sterilization: our experience by* Fedeli et al.). Women who have undergone sterilization performed negligently are entitled to recover damages for wrongful conception, negligence, and wrongful birth. The authors also consider the issue of female sterilization in minors. Primary importance of thorough and complete communication of information were emphasized. The surgical sterilization procedure is generally safe and highly effective. The setting, the surgeon's experience, the country's economic development, and the woman's preference are the elements that determine the decision regarding the surgical method. The authors emphasized that some techniques present a greater risk of failure and expose the surgeon to malpractice litigation.

The “opinion” article authored by Cestonaro and Aprile (*Legal, deontological, and ethical basis of the physician–minor patient care relationship)* concerns the relationship between healthcare professionals and minors. There are not an agreement on the age at which a minor can be deemed competent for decision-making, and also about the identification of the age at which minors can consent to medical treatments without parental consent. The situation are very dissimilar among countries. The authors made a review the legal, deontological, and ethical references that concern the doctor–minor patient relationship in Italy (Law n. 194 of 1978, Article 609-quater of the Italian penal code, Law n. 219 of 2017, the physician code of deontology, and the ethical point of view). The author emphasized the principle of respect for autonomy, and the right to make choices and take actions based on personal values and beliefs. When the minor grows up, a direct relationship with the clinician becomes established, and confidential care issues emerge. It has been highlighted the importance of confidential care for adolescents to encourage access to care that impacts the wellbeing of the minor, such as the decision to use emergency contraception. The fear of a lack of confidentiality, may discourage adolescents from seeking health counseling, even when dealing with sensitive issues such as contraception and sexual activities. If healthcare professionals build trust both with parents and the minors to facilitate parental acceptance of confidential care it may achieve more success.

The “perspective” article authored by Scendoni et al. (*Over-the-counter emergency contraception in Italy: ethical reflections and medico-legal issues*) concern the ethical and medico-legal problems connected with emergency contraception. Emergency contraception remains a complex issue with many scientific, legal, ethical, and social implications. The authors highlights the incompleteness of the scientific reconstruction on the effects of emergency hormonal contraceptives and the potential risks associated with the decision to exempt the supply of over-the-counter drugs from the general rules of healthcare. The authors affirm the need for a comprehensive scientific understanding of the effects of emergency hormonal contraceptives and express concerns about deviating the supply of over-the-counter drugs from the established norms of healthcare. Various ethical and medico-legal issues are addressed, with a specific focus on underage women whose sexual and reproductive health necessitates proactive intervention without medicalizing their choices.

The Research Topic presents original scientific results, knowledge reviews, opinions, and discussion perspectives related to the topic of reproductive medical care in minors. Some contributions delve into the ethical, deontological, and medico-legal issues connected with these procedures when dedicated to minors. Readers can find valuable information and interesting references on the topic in the contributions contained in the Research Topic, providing a starting point for further study.

In particular, we believe it is important to promote research activities aimed at obtaining useful evidence regarding risk management in the field of adolescent sexual health. This type of study could reveal new correlations that are valuable for more systemic prevention. A second area of research that we aim to develop pertains to the modulation of sexual behavior through social media in various pediatric age groups. Without awareness of these dynamics, the educational efforts implemented by schools and families can easily not only be questioned but also become ineffective.

## Author contributions

MC: Writing—original draft, Writing—review & editing. TV: Writing—original draft, Writing—review & editing.

## Footnotes

^1^Available online at: https://www.astho.org/topic/brief/current-trends-in-adolescent-sexual-reproductive-health/.

